# Elucidating the Quenching Mechanism in Carbon Dot-Metal Interactions–Designing Sensitive and Selective Optical Probes

**DOI:** 10.3390/s21041391

**Published:** 2021-02-17

**Authors:** Farah Noun, Evelyne Anastasia Jury, Rafik Naccache

**Affiliations:** 1 Department of Chemistry and Biochemistry, Concordia University, Montreal, QC H4B 1R6, Canada; farah.noun@mail.concordia.ca (F.N.); evelyneajury@gmail.com (E.A.J.); 2 Quebec Centre for Advanced Materials, Department of Chemistry and Biochemistry, Concordia University, Montreal, QC H4B 1R6, Canada

**Keywords:** carbon dots, sensors, metals, fluorescence, quenching mechanism, mercury

## Abstract

Overexposure to metals has significant adverse effects on human and animal health coupled with nefarious consequences to the environment. Sensitive tools to measure low contaminant levels exist, but often come at a high cost and require tedious procedures. Thus, there exists a need for the development of affordable metal sensors that can offer high sensitivity and selectivity while being accessible on a global scale. Here, carbon dots, prepared in a one-pot synthesis using glutathione and formamide, have been developed as dual fluorescent metal sensing probes. Following extensive characterization of their physico-chemical properties, it is demonstrated that dual fluorescence can be exploited to build a robust ratiometric sensor with low-ppb detection sensitivity in water. This investigation shows that these optical probes are selective for Pb^2+^ and Hg^2+^ ions. Using steady-state and dynamic optical characterization techniques, coupled with hard and soft acid-base theory, the underlying reason for this selective behavior was identified. These findings shed light on the nature of metal-carbon dot interactions, which can be used to tailor their properties to target specific metal ions. Finally, these findings can be applicable to other fluorescent nanoparticle systems that are targeted for development as metal sensors.

## 1. Introduction

Over the past decade, metal detection and sensing methods have garnered significant attention owing to the ever-growing concerns related to environmental metal contamination [1,2,3,4]. With their high surface area-to-volume ratios, their tunable fluorescence and versatile surface chemistries, which allow for their incorporation in different environments and matrices, nanoparticles (NPs) have been touted as an interesting option in metal sensing applications [5,6,7,8,9,10,11]. More specifically, carbon dots (CDs) possess many advantageous properties to serve as metal nanosensors, including their chemical versatility, hydrophilicity, photostability and low chemical and cytotoxicity [11,12,13,14,15,16,17,18]. Previous works have evidenced excellent CD sensitivity in detecting metals such as Hg^2+^ [19,20,21,22,23,24], Fe^3+^ [25,26] and Pb^2+^ [27,28], among others [13,29,30,31]. 

Despite widespread interest, there remains a lack of understanding related to the fundamental chemistry of the metal-CD interactions central to sensing applications. Although some theories concerning these interactions have been postulated [23,24,26,27], many questions remain unanswered. For instance, the quenching mechanism remains completely overlooked, or often only briefly discussed without offering compelling data. Previous publications on metal sensing CDs have suggested mechanisms related to unspecified non-radiative electron transfer from the CD to the metal cation, with some studies suggesting coordination of the metal to certain functional groups on the CD surface, such as hydroxyl groups. Yet, many of these claims remain unsupported by convincing evidence, and, in some cases, the suggested quenching mechanism actually disagrees with the data provided. 

Fluorescence-based detection of metal cations depends on fluorescence quenching through metal-CD interactions which can be categorized as static or dynamic quenching. In static quenching, a non-emissive complex is formed between the CD and the metal cation causing a previously emissive state to return to the ground state without the emission of a photon [32,33]. An increase in quencher concentration will typically result in changes in the absorbance spectra with no changes in fluorescence lifetimes for a static quenching mechanism. Moreover, an increase in system temperature will cause decreased quenching. Dynamic quenching, often referred to as collisional quenching, occurs as a result of collisions or close-contact between the metal cation and the excited-state CD, which result in an energy transfer that returns the CD to the ground state without the emission of a photon [32,33]. An increase in quencher concentration will typically result in changes in fluorescence lifetimes with no changes in the absorbance spectra for a dynamic quenching mechanism. Moreover, an increase in system temperature will produce increased quenching [32,33]. Dynamic quenching includes several mechanisms such as photo-induced electron transfer (PET), Förster resonance energy transfer (FRET) and surface energy transfer (SET) [33]. Additionally, inner filter effect (IFE) is included as a concentration-based trivial, or non-molecular, quenching mechanism [32,34].

PET is a redox-type quenching mechanism in which a radical anion and cation are formed as a charge-separated complex when a source of light excites a particle and induces the transfer of an electron from a donor to an acceptor. As such, the presence of radical ions can be observed in a PET mechanism through ultra-fast spectroscopy [32,33,35]. In FRET, energy from an excited state fluorophore is transferred to a ground-state quencher without the emission of a photon [36]. This energy transfer depends on the overlap between the donor emission spectrum and the acceptor absorption spectrum, the distance between the donor and acceptor, as well as their relative orientations [32,33,37]. FRET involves energy transfer through dipole-dipole interactions between a donor and an acceptor, thus only occurs within a range of 1 to 10 nm [32,33]. SET is a phenomenon similar to FRET, but based on dipole-surface interactions, that has mainly been observed between a fluorescent dipole and a metal nanoparticle or surface [33,38]. This interaction differs in that the metallic surface presents isotropic dipole vectors [38], so the transfer occurs due to a dipole-surface resonance mechanism with a much slower decay rate [39]. In this manner, SET results in an effective transfer over larger distances between acceptor and donor than FRET, by more than double the latter [39]. Inner filter effect (IFE) is a trivial quenching mechanism which can occur in highly concentrated samples. In such a sample, there is a chance that the excitation photon or an emitted photon from a fluorescent particle will be intercepted and absorbed by a quencher within the sample before reaching the detector [33]. As such, there needs be spectral overlap between the emission and absorbance spectra of the CD and the interfering species. IFE does not affect fluorescence lifetimes, and the use of dilute solutions can help reduce the occurrence of this effect [33,40,41,42].

**Scheme 1:**
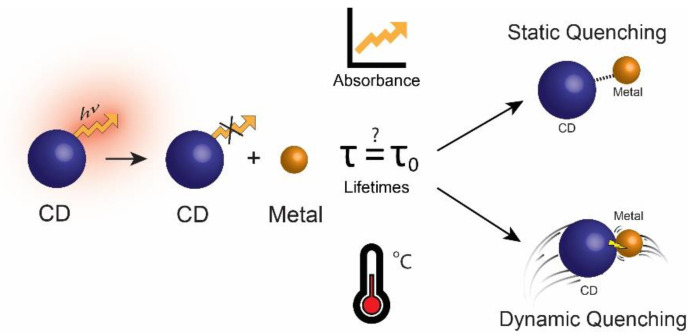
Metal quenching is investigated using steady-state (absorbance and fluorescence), as well as dynamic (fluorescence lifetime) techniques in order to elucidate an understanding of the mechanism and the role of the surface chemistry of the optical probe.

With so many mechanisms potentially influencing fluorescence-based detection, it is essential that the fundamental processes governing CD metal quenching and the surface groups, which play a role in this process, be definitively identified in order to develop concrete metal sensing tools that can translate to commercial applications. In this work, formamide-glutathione CDs (FG-CDs) are subjected to quenching assays with mercury (II) and lead (II) cations. Both steady-state and dynamic optical characterization techniques are utilized to provide a better understanding of metal-CD interactions (see Scheme 1). The quenching mechanism is identified for these CDs and a theory is presented to elucidate the surface functional groups involved in these metal-CD interactions [32,33]. This greater understanding of how quenching occurs will, in turn, allow CDs to be further optimized to obtain better selectivity and sensitivity.

## 2. Materials and Methods

### 2.1. Chemicals and Reagents 

Glutathione, lead (II) chloride, mercury (II) chloride, and ethanol were purchased from Sigma Aldrich. Formamide, acetone and LC-MS water were purchased from Fisher Scientific. All reagents used were of analytical grade and did not necessitate additional purification prior to use.

### 2.2. Synthesis of FG-CDs

To synthesize the FG-CDs, 0.334 g of glutathione dissolved in 10 mL of formamide were used to form a 0.1 M solution. The solution was sonicated until it was completely clear before being transferred to a 35 mL microwave vial. The vial was then heated for 5 min at 180 °C in a CEM Discover SP microwave reactor. The resulting dark green solution was dialyzed for 5 days with a 3.5–5.0 kDa MWCO cellulose-ester membrane (Spectra/Por^®^ 6 RC—Spectrum Laboratories), changing the water every 24 h to remove small, water-soluble and membrane-permeable impurities. The solution was then concentrated down in an 80 °C oven before being passed through a 0.22 μm nylon-filter to remove large aggregates within the solution. Next, the sample was subjected to an organic acetone wash, followed by 2 organic ethanol washes and a final acetone wash with centrifugation at 10,000× *g* for 5 min following each wash to remove any impurities left behind by dialysis. Finally, the CDs were dried at 80 °C before being re-dispersed in water at a known concentration. 

### 2.3. Transmission Electron Microscopy (TEM)

Grids were prepared by pipetting 2 µL of a 5.0 mg/mL CD dispersion onto a 200 mesh Formvar/carbon coated copper grid (3 mm in diameter) followed by evaporation of the solvent. The TEM images were collected using a Jeol JEM-2100F microscope operating at 100 kV. The Fiji imaging software was used for image processing and to determine the size of the CDs [43].

### 2.4. X-ray Photoelectron Spectroscopy (XPS)

XPS spectra were obtained using a Thermo Scientific K-Alpha X-ray Photoelectron Spectrometer equipped with an Al K alpha source gun in standard lens mode, a CAE analyzer mode using 50.0 eV pass energy with a 0.100 eV energy step size and a 400 mm spot size. Each analysis was carried out in triplicates using 10 runs for each scan. The averages for both the survey and high-resolution scans were plotted. The Thermo Scientific Avantage Data System software was used for data collection and analysis.

### 2.5. Fourier Transform Infrared (FTIR)

FTIR spectra were acquired using a Thermo Scientific Nicolet iS5 equipped with iD5 ATR accessory. Dried powder CD samples were used as is, while 1 µL of the aqueous CD dispersions was allowed to evaporate to dryness, at room temperature, for 15 min prior to spectrum acquisition. A total of 64 scans, at a resolution of 0.4 cm^−1^, were obtained on a laminate-diamond crystal window, using the following instrument settings: a DTGS KBr detector set to a gain of 1, an optical velocity of 0.4747 cm/s and an aperture of 100. The Thermo Scientific Nicolet Omnic 9 software was used for all data processing.

### 2.6. Zeta Potential 

Zeta potential (surface charge) measurements were obtained using a Malvern Zetasizer Nano-S. All experiments were carried out using a disposable folded capillary cell (Malvern). For each sample, experiments were repeated three times at 25 °C using the average of 13–15 runs each. The concentration of the samples was ~0.35 µg/mL in LC-MS water. Measurements were conducted using approximately 0.7 mL of the solution. Data were analyzed using the software supplied with the instrument.

### 2.7. Absorbance 

UV-Vis absorbance spectra of the aqueous CD dispersions were collected using a Cary 5000 Series UV-Vis-NIR Spectrophotometer from Agilent Technologies. The scans were acquired over a range of 200–800 nm in a 1-cm quartz cuvette at a speed of 600 nm/s, 1 nm resolution and a 2-nm bandwidth. A Peltier Temperature Controller from Agilent Technologies was used to heat and cool the sample cell for temperature experiments. Data collection and processing were performed using the Agilent Cary Eclipse Scan software package. 

### 2.8. Fluorescence 

The fluorescence spectra of the aqueous CD dispersions were recorded using a Cary Eclipse Fluorescence Spectrophotometer from Agilent Technologies. To minimize potential inner-filter effects, sample concentrations were adjusted to an absorbance value up to 0.4 A.U. prior to the collection of the fluorescence spectra [44,45]. Fluorescence spectra were acquired in a 1-cm quartz cuvette, using an excitation slit width of 2.5 nm, an emission slit width of 5 nm with a PMT voltage of 600 V. Data collection and processing were performed using the Agilent Cary Eclipse application software.

### 2.9. Fluorescence Lifetimes 

The EasyLife X fluorescence lifetime system (Optical Building Blocks Corporation) was used for acquisition of the CD’s fluorescence lifetimes. Measurements were conducted in a 1 cm quartz fluorescence cuvette, using a 368 nm pulsed picosecond LED excitation source. The collection parameters were as follows: an emission slit width of 1.5 mm, 500 channels, 0.25 s integration time and an average of 3 readings for each sample. A bi-exponential decay was determined with a random collection mode to account for any potential photobleaching. Moreover, logarithmic collection steps were used to obtain more data points at the time of the pulse and at the beginning of the decay. The OBB EasyLife X software was used to collect and analyze the data. 

### 2.10. Quenching Assays 

All solutions were prepared in LC-MS water immediately prior to performing quenching assays. Concentrated CD dispersions were sonicated and used to produce 8 mL of 35 μg/mL FG-CDs. UV-Vis and Fluorescence measurements of the FG-CD sample were taken at increasing concentrations of mercury (II) ions. Prior to spectroscopic measurements, the samples were stirred using a magnetic stirrer for approximately 20 s following the addition of the metal salt solution. 

## 3. Results and Discussion

In this study, the FG-CDs were used in metal quenching assays with mercury (II) cations in order to investigate the quenching mechanism occurring and glean information regarding the role of the functional groups responsible for the metal-CD interaction. These CDs have been previously characterized and successfully used for the detection of Pb (II) [16,18,28]. TEM imaging (Figure S1) shows that these CDs are quasi-spherical with an average size of 7.7 ± 1.5 nm, and a relatively broad size distribution. Their surface chemical groups were analyzed using FTIR (Figure S2) and XPS (Figure S3) with the results being summarized in Table S1. The FTIR spectrum exhibits broad stretches ranging from 3000 to 3500 cm^−1^ associated with N-H bonds in amines/amides and O-H bonds from hydroxyl/carboxylic acid groups on the CD surface. The presence of amide functional groups is indicated by the strong amide C=O stretch at 1645 cm^−1^. This is further supported by the presence of C-N amide stretches at 1307 cm^−1^ and 1386 cm^−1^. A stretch at 1583 cm^−1^ may also indicate the presence of C=C and C=N functional groups [16].

These findings were also confirmed by the XPS data collected. The survey spectrum in Figure S3A revealed that the elemental composition of the CDs was broken down to 53.1% carbon, 26.1% oxygen, 17.4% nitrogen and 3.4% sulfur [16]. Deconvolution of the Carbon (C1s), nitrogen (N1s), oxygen (O1s) and sulfur (S2s) binding energies revealed the presence of the following functional groups within the CDs. First, deconvolution of the C1s (shown in Figure S3B) indicated binding energies at 288.68 eV, 286.89 eV and 285.58 eV associated with C-C/C=C, C-O and C=O/C=N functional groups, respectively. The oxygen O1s binding energies (Figure S3C) uncovered the presence of C-OH/C-OC and C=O functional groups at 533.26 and 532.02 eV, respectively. The deconvoluted N1s binding energies in Figure S3D at 402.71 and 400.58 eV were ascribed to NH_2_/pyrrolic N and graphitic N, respectively. Finally, for S2p (Figure S3E), a thiol functional group shows a binding energy at 165.51 eV, while two thiophene groups were linked to binding energies at 164.14 and 163.38 eV [16]. 

**Figure 1:**
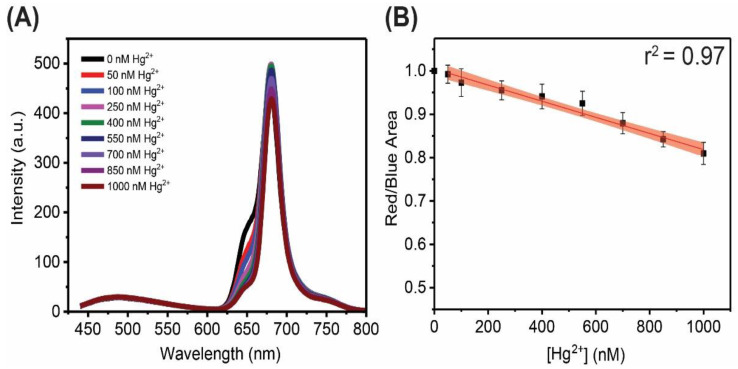
Results of a FG-CD quenching assay with mercury (II) ions showing (**A**) the fluorescence spectra with changes in the 650 and 680 nm peak, while the 485 nm peak remains unaffected by the presence of mercury (II) cations and (**B**) the linear plot of the decreasing overall R/B area ratio showing a ~20% decrease in red fluorescence with an r^2^ = 0.97.

FG-CDs possess dual fluorescence that is attributed to different emissive states within the dot. The blue fluorescence centered at 480 nm is believed to arise from the core of the particle, and is thus largely unaffected by the CD’s environment, while the red fluorescence centered at ~680 nm is ascribed to surface emissive states, which are easily perturbed by the dots’ external environment [14,16,18,46]. Following the addition of metal cations, fluorescence quenching is indeed observed with increasing concentration of mercury (II) ions, as shown in Figure 1A. No significant changes in the blue fluorescence band are noted; however, the dots’ red fluorescence (centered at ~680 nm with a ~650 nm shoulder) is quenched by the addition of the metal cation [14,16,18,46]. Using the ratiometric approach, quenching is calculated by measuring the change in the integrated area under the curve of the red band over that of the blue counterpart. The red fluorescence decreases while the blue component remains relatively unchanged, resulting in an overall decrease in the Red/Blue ratio (R/B ratio). This unique dual fluorescence permits the quantification of metal cations in solution through a robust ratiometric method in lieu of an intensity-based approach relying on a single fluorescence band. The advantage of the former approach is noted as having an internal self-reference. Figure 1B shows a plot of the decrease in the red/blue (R/B) ratio with a linear trend (r^2^ = 0.97) and a confidence interval of 95% shown as the red shaded area. The R/B ratio decreases to 80% of its original value (which has been normalized to 1) following the addition of 1000 nM of Hg^2+^. 

It should be noted that although a constant decrease in the intensity of the 650 nm shoulder in the fluorescence spectrum is observed, the principal peak at 680 nm initially increases in intensity as the metal concentration increases. This trend is maintained up to a concentration of 250 nM Hg^2+^ after which it subsequently decreases for the remainder of the quenching assay. This behavior is likely indicative of an energy transfer, which occurs as a result of the quenching of the 650 nm emissive state, at low Hg^2+^ concentrations. It is possible that this occurs through binding of the metal cations to the surface functional groups responsible for that emissive state. Once these states are saturated, then quenching of the 680 nm peak at higher metal ion concentrations follows. Despite this observation, the overall R/B ratio consistently decreases with increasing Hg^2+^ ions. The LOD was calculated using the standard 3σ error divided by the slope of the linear trend and was found to be equal to ~104 nM (or 28 ppb). 

**Figure 2:**
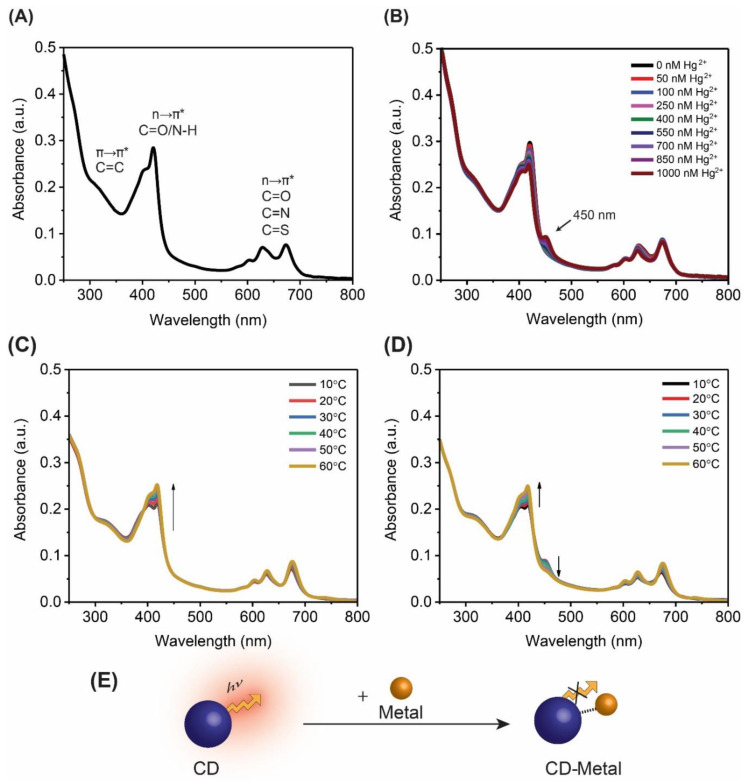
Absorbance spectra for FG-CDs (**A**) alone, (**B**) showing a new peak at ~450 nm in the presence of Hg^2+^. Absorbance spectra of FG-CDs showing changes as a function of increasing temperatures (**C**) with 0 nM of Hg^2+^ and (**D**) with 1000 nM of Hg^2+^. (**E**) Graphical illustration of CD fluorescence quenching upon binding to a metallic cation.

In a series of experiments, the absorbance spectra of the system were first collected at increasing concentrations of metal cations. Figure 2A shows that the FG-CDs alone possess several absorbance peaks. There is a weak absorption band near 300 nm, which corresponds to the π→π* transition of the C=C sp^2^ domains in the carbon dot core [47]. The most intense absorption occurs between 350 and 425 nm, with a maximum at 420 nm associated with the n→π* transition of the carbonyl and/or amine groups within the CDs. The last transition is the n→π*, which occurs between 600–700 nm, is ascribed to C=O, C=N and C=S functional groups in the aromatic carbon network [40,41].

Figure 2B depicts changes in the absorbance spectra of FG-CDs upon the addition of mercury (II) cations. In the presence of Hg^2+^, the absorbance peak at 420 nm, the same wavelength used for excitation in the fluorescence spectra, gradually decreases in intensity as metal concentration increases. This correlates with the overall decrease in fluorescence intensity observed in Figure 1. However, what is most notable in Figure 2B is the appearance of a new band at ~450 nm, exclusively observed with the addition of mercury (II) cations. The theory on static quenching describes changes in the absorbance spectra related to the formation of a new CD-metal complex with its own unique absorbance band, which explains the appearance of this peak in the FG-CD system. Moreover, this peak increases in intensity with greater concentrations of mercury (II) ions, thus supporting the idea that the availability of mercury (II) ions promotes the formation of a CD-mercury complex. Dynamic quenching typically shows no such changes in the absorbance spectra and, based on observations from this study, it does not appear to be responsible for the observed behavior. Similar changes in the fluorescence and absorbance spectra of these CDs have previously been observed in the presence of Pb^2+^ ions, with the absorbance spectra showing the appearance of a new peak around ~475 nm [28]. For the purposes of this study, another quenching experiment was performed with Pb^2+^ to confirm these results. It was found that Pb^2+^ quenched ~20% more than Hg^2+^ and had a lower LOD at 81 nM (or 22 ppb). These results are shown in Figure S4. These changes in the absorbance spectra indicate that a static quenching mechanism is likely occurring for this CD system with these two metal cations.

In order to further investigate the quenching mechanism, the fluorescence lifetimes of the sample were measured at different concentrations of mercury (II) cations. Table 1 summarizes the two lifetime components for FG-CDs: a short component of 0.4 ns and a long component of 5.6 ns. The long component is believed to be associated with the blue fluorescence, stemming from the core of the particle, and the short component with the red counterpart, originating from the surface of the dot [28]. Despite the decrease in red fluorescence emission, both lifetimes remain unchanged regardless of mercury (II) concentration further supporting a static quenching mechanism. Similarly, the lifetime experiment was repeated for these CDs in the presence of Pb^2+^ (Table S2), where the same conclusion was reached.

Finally, temperature studies were performed by tracking changes in the absorbance spectra of FG-CDs at various temperatures in the presence of 1000 nM Hg^2+^. In a statically quenched system, it is expected that an increase in temperature will destabilize the formed complex between the CD and metal ion due to greater energy within the system. This leads to the breaking of the non-emissive formed complex, thus a reduction of quenching [32,33]. Figure 2C,D shows the changes in the absorbance spectra of FG-CDs as a function of temperature both in the presence and in the absence of mercury (II) ions. The control FG-CDs (in the absence of the metal ions) in Figure 2C, display an increase in the 420 nm band as a function of increasing temperature, which correlates with the enhancement of the red fluorescence emission reported for these CDs at increasing temperatures [16]. This could be attributed to the promotion of FRET pathways within the sample at higher temperatures [42,48]. FG-CDs meet the spectral overlap requirements of FRET, wherein there is an overlap of their absorbance and emission spectra in several regions [33,42]. Increased particle movement and collisions due to higher temperatures may help the system meet the 1–10 nm distance requirement of FRET [33,49]. These energy transfers typically flow from higher to lower energy states, thus promoting the red fluorescence emission over that of the blue. Though other mechanisms could be occurring between the CDs, the system does not meet one or more of their requirements such as: the presence of a metallic surface or nanoparticle (SET) or temperature independence (PET and IFE) [34,50]. However, further steady-state and dynamic optical studies are required to confirm this hypothesis. In the same system and in the presence of Hg^2+^, shown in Figure 2D, the 450 nm peak associated with the CD-mercury complex decreases in intensity with increasing temperature. This indicates the de-stabilization and the breaking apart of the formed CD-mercury complex at higher temperatures, which results in the loss of its absorbance peak at 450 nm. As such, it can be concluded that a static quenching mechanism is occurring with these CDs in the presence of Hg^2+^. 

For systems with an exclusively static or exclusively dynamic quenching mechanisms, Stern-Volmer plots will present a linear trend. Differentiating between the two types of mechanisms will require analysis of the absorbance spectra and fluorescence lifetime measurements. However, a deviation from linearity, such as in Figure S5 where there is an initial downward trend followed by an upward trend, typically indicates that the system could be experiencing more than one quenching mechanism or could be unsuitable for a classical Stern-Volmer equation [32,34,51]. The initial downward deviation, which begins at the 250 nM concentration, could be attributed to the presence of 2 or more emissive states in the CD and the complex energy transfers occurring between them. As previously mentioned, there seem to be at least 2 emissive states, which are both binding sites for metallic cations. While the fluorescence intensity of one emissive state decreases with the addition of metal cations, the second emissive state, to which the metal cations have a lesser affinity, sees an initial increase in fluorescence intensity up to a concentration of 250 nM. After saturation of the first binding site, the second binding site begins to bind metal cations, causing this emissive state to be quenched as well. Considering the differences in affinity for metal cations, there may be two or more separate quenching rate constants in action. Such heterogeneity in fluorescent species is known to cause downward curves in a classical Stern-Volmer plots [32,34]. 

Due to the changes observed in the absorbance spectra, the results of the temperature study as well as the lack of changes in the fluorescence lifetimes (all indications of static quenching), none of the data collected suggests a dynamic quenching mechanism. Thus, although still possible, it is unlikely that dynamic quenching between the CD and metal cations is contributing to the subsequent upward curve observed in the plot. Moreover, inner-filter effect is avoided with the use of low concentration solutions, thus is also not likely to be a significant contributing factor to this upward trend. This is supported by the results of the temperature study, which show clear changes in absorbance with changing temperatures, as temperature does not affect sample concentration [34]. 

However, various energy transfer processes could be occurring within or between the CDs that could contribute to the deviation from linearity observed. One such process is FRET, a type of dynamic quenching which is postulated to be the cause of the increase in red fluorescence with increasing temperatures, a process that occurs between the CDs with and without the presence of metal quenchers [16]. Another possible process is the unspecified energy transfer which occurs upon binding of the first emissive state to metal cations, wherein energy is transferred to a second emissive state, causing an increase in this second fluorescence emission upon binding. Both these processes could contribute to the non-linearity observed in Figure S5. 

Identifying the quenching mechanism alone is insufficient to accede to a fundamental understanding of CD-metal interactions. Further knowledge concerning the activity of the functional groups, which play a role in these interactions, is necessary for the design of more sensitive and selective CD metal sensors. For this purpose, these CDs were tested using a wide selection of metal ions with varying electronic properties including oxidation state, number of valence electrons, ionic size and ionic charge. The results plotted in Figure S6 show that quenching is only observed with lead (II) and mercury (II) ions. The notable similarities between lead and mercury are that both ions possess a +2 charge but, unlike the metal ions tested bearing a similar charge, these ions had a significantly larger ionic radius. As such, the charge densities of the metal cations were studied in order to glean a better understanding of the observed trends. In Table 2, it can be seen that lead (II) and mercury (II) ions have significantly lower charge densities than the other metal ions investigated in this work [52]. Therefore, the charge density of an ion seems to be an important factor in predicting whether the ion will bind to the FG-CDs.

For this reason, the surface charge of the FG-CDs was determined via zeta potential measurements. They were found to have an overall negative surface charge of −22.3 mV; however, positive charges were also detected on the CD surface. Previous characterization using FTIR spectroscopy and XPS has shown the presence of both positively- and negatively-charged surface functional groups for FG-CDs, including thiols, amines and carboxylic acids [16,18,28]. Consequently, it is likely that these positive surface charges may be resulting in a repulsion with the metallic cations that possess a higher charge density. The charge densities of mercury (II) and lead (II) ions may be low enough to minimize repulsion allowing them to bind to negatively charged functional groups on the CD surface. Among these functional groups, thiols and thiophenes are of particular interest due to the exceptionally strong interaction of sulfur-containing compounds with mercury and lead [53,54,55]. In the Hard and Soft Acid-Base (HSAB) theory, a hard ion is described as one with a low polarizability, a large charge density and a small ionic radius; these ions tend to form ionic type bonds. Conversely, a soft ion is one with a high polarizability, a small charge density and a large ionic radius; these ions tend to form covalent type bonds. Mercury and lead are categorized as soft and borderline acids, respectively, which bind more strongly to soft bases, such as the thiols and thiophenes which are present on the FG-CD surface [56,57,58]. Lead in particular is shown to have an especially strong affinity to soft, sulfur-containing ligands compared to a number of other soft and borderline acids which correlates with its stronger quenching of FG-CDs observed in Figure S6 [56]. Therefore, the quenching of FG-CDs by mercury and lead is very likely occurring through a static quenching mechanism where these soft and borderline acid cations bind with surface sulfur-containing groups on the CDs, while avoiding electrostatic repulsion from positively charged surface functional groups due to their lower charge densities.

FG-CDs possess two main sulfur-containing groups namely thiols and thiophenes and both are soft ligands able to bind with soft metallic cations [16]. However, due to electron delocalization in the thiophene ring, it can be classified as softer than the thiol groups. As such, the soft metallic cations would initially bind to these sites until saturation, after which binding to the thiol groups will occur. This may explain the trend observed in Figure 1, wherein one emissive state initially increases while the other decreases in intensity. The thiol and thiophene groups may represent two red emissive states, where the thiophene state is quenched upon binding with the metal cation and energy from this state is then transferred to the emissive thiol state. With increasing cation concentration, the thiol states in turn begin binding to the cations and are subsequently quenched leading to a decrease in the overall red fluorescence emission. 

Tailoring the abundance of these sulfur-containing groups in the CDs could potentially increase their affinity for lead and mercury, while modifying other surface functional groups could allow the binding of different metal ions by eliminating electrostatic repulsion. Nevertheless, it is possible that other functional groups may also bind these metallic cations or be otherwise responsible for these metal-CD interactions. However, to date, no evidence has been shown to support this.

## 4. Conclusions

With growing concerns over the environmental and health impacts of metal contamination, this work aimed to further the knowledge of metal-CD interactions to give researchers the necessary understanding to design more sensitive metal sensing CD systems. For this purpose, steady-state and dynamic optical characterization techniques were used to elucidate the quenching mechanism occurring and the functional groups responsible for this phenomenon. Thus, it was determined that FG-CDs could detect lead (II) and mercury (II) cations with LODs of 22 and 28 ppb, respectively, through a static quenching mechanism. It was further theorized that the metal-CD interaction stems from the electrostatic attraction of the positively charged metal cations and the negatively charged thiols and thiophenes on the surface of the CD. Moreover, the selectivity observed (wherein FG-CDs only detect mercury and lead out of a number of various metal cations) is seemingly due to the lower charge density of these two metals in comparison to all other cations tested, which may be experiencing electrostatic repulsion from positively charged functional groups on the CD surface. 

## Figures and Tables

**Table 1:** Fluorescence lifetimes of FG-CDs at increasing concentrations of mercury (II). ions.

[Hg^2+^]	Lifetime 1 (ns)	±	Lifetime 2 (ns)	±	χ^2^
0 nM	0.4	2.9 × 10^−3^	5.5	1.1 × 10^−2^	1.1
400 nM	0.4	1.1 × 10^−2^	5.5	2.2 × 10^−2^	1.3
1000 nM	0.4	7.1 × 10^−3^	5.6	2.3 × 10^−2^	1.1

**Table 2:** Charge, ionic radii and charge densities of tested metal cations. Values obtained from [52].

Metal Ion	Charge	Ionic Radius (Å)	Charge Density (C·mm^−1^)
**Fe^3+^**	3+	0.78	232
**Pb^2+^**	2+	1.19	32
**Cu^2+^**	2+	0.87	116
**Co^2+^**	2+	0.88	108
**Hg^2+^**	2+	1.02	49
**Ni^2+^**	2+	0.83	134
**Cr^3+^**	3+	0.52	261
**Gd^3+^**	3+	0.94	91
**Dy^3+^**	3+	0.91	99

## Data Availability

Data is contained within the article or supplementary material. Any additional data concerning the work in this study are available on request from the corresponding author.

## Supplementary Materials

The following are available online at https://www.mdpi.com/1424-8220/21/4/1391/s1, Table S1: Summary of characterization for FG-CDs showing (A) the functional groups observed from FTIR analysis (B) the functional groups observed from XPS analysis and (C) the elemental composition of the dots, Table S2: Fluorescence lifetimes of FG-CDs at increasing concentrations of lead (II) ions, Figure S1: TEM image of FG-CDs with in-laid size distribution plot showing an average size of 7.7 ± 1.5 nm, Figure S2: FTIR spectrum of FG-CDs showing the presence of amide and carboxyl stretches as well as N-H and O-H functional groups, Figure S3: (A) XPS survey spectrum of FG-CDs showing binding energies of C1s, N1s, O1s and S2p. Spectra of deconvoluted binding energies reveal (B) a maximum for C1s at 286.08 eV, (C) a maximum at 400.08 eV for N1s (D) a maximum at 532.08 eV for O1s and (E) for S2p a maximum at 165.08 eV, Figure S4: (A) Absorbance spectra for FG-CDs showing a new peak at ~475 nm in the presence of Pb^2+^. (B) Fluorescence spectra for FG-CDs in the presence of Pb^2+^. (C) The linear plot of the decreasing overall R/B area ratio showing a ~40% in red fluorescence with an r^2^ = 0.98, Figure S5: (A) Stern-Volmer plot displaying the linear equation for FG-CDs and Hg^2+^ with ksv = 2.14 × 10^−4^ and r^2^ = 0.95 (B) Stern-Volmer plot displaying the linear equation for FG-CDs and Pb^2+^ with ksv = 4.88 × 10^−4^ and r^2^ = 0.94, Figure S6: R/B area ratio of FG-CDs with 1000 nM of various metallic cations showing comparison of quenching effectiveness.
