# Supplementary material for: Elucidating the Quenching Mechanism in Carbon Dot-Metal Interactions–Designing Sensitive and Selective Optical Probes
Source: Sensors (Basel). 2021 Feb 17;21(4):1391. doi: 10.3390/s21041391 (PMC7922893; doi:10.3390/s21041391)
Supplement: Supplementary file 1 [file sensors-21-01391-s001.pdf]

Supporting Information

# Elucidating the Quenching Mechanism in Carbon Dot-Metal Interactions–Designing Sensitive and Selective Optical Probes

**Farah Noun, Evelyne Anastasia Jury and Rafik Naccache**

**Table S1**: Summary of characterization for FG-CDs showing **(A)** the functional groups observed from FTIR analysis **(B)** the functional groups observed from XPS analysis and **(C)** the elemental composition of the dots.

## (A) FTIR Analysis

| Wavenumber ($cm^{-1}$) | Type of vibration | Type of bond | Functional group |
|---|---|---|---|
| 1307 & 1386 | Stretch | C-N | amide/amine |
| 1583 | Stretch | C=N | - |
| 1583 | Stretch | C=C | Aromatic |
| 1645 | Stretch | C=O | Amide |
| 3000-3500 | Stretch | N-H | amide/amine |
| 3000-3500 | Stretch | O-H | hydroxyl/carboxylic acid |

## (B) XPS Analysis

| Peak | Binding energies (eV) | Type of bond | Functional group |
|---|---|---|---|
| C1s | 285.58 | C=O/C=N | amide/carboxylic |
| | 286.89 | C-O | - |
| | 288.68 | C-C/C=C | - |
| N1s | 400.58 | C=N/C-N | graphitic nitrogen |
| | 402.71 | $NH_2$ | pyrrolic nitrogen |
| O1s | 532.02 | C=O | amide/carboxylic |
| | 533.26 | C-OH/C-OC | - |
| S2p | 163.38 | $C_4S$-H | thiophene |
| | 164.14 | $C_4S$-H | thiophene |
| | 165.51 | C-S-H | thiol |

## (C) Elemental Analysis

| Element | Percent Composition |
|---|---|
| Carbon | 53.10% |
| Oxygen | 26.10% |
| Nitrogen | 17.40% |
| Sulfur | 3.40% |

**Table S2:** Fluorescence lifetimes of FG-CDs at increasing concentrations of lead (II) ions.

| $[Pb^{2+}]$ | Lifetime 1 (ns) | ± | Lifetime 2 (ns) | ± | $\chi^2$ |
|---|---|---|---|---|---|
| 0 nM | 0.8 | 2.8E-03 | 5.7 | 4.5E-03 | 1.2 |
| 400 nM | 0.8 | 4.1E-03 | 5.9 | 8.1E-03 | 1.1 |
| 1000 nM | 0.8 | 3.3E-02 | 5.7 | 3.5E-02 | 1.1 |

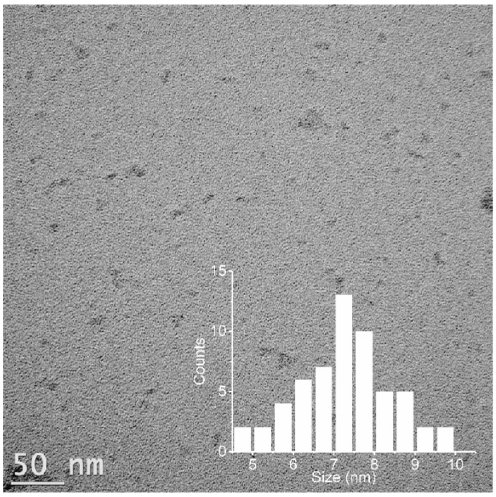


**Figure S1:** TEM image of FG-CDs with in-laid size distribution plot showing an average size of 7.7 ± 1.5 nm.

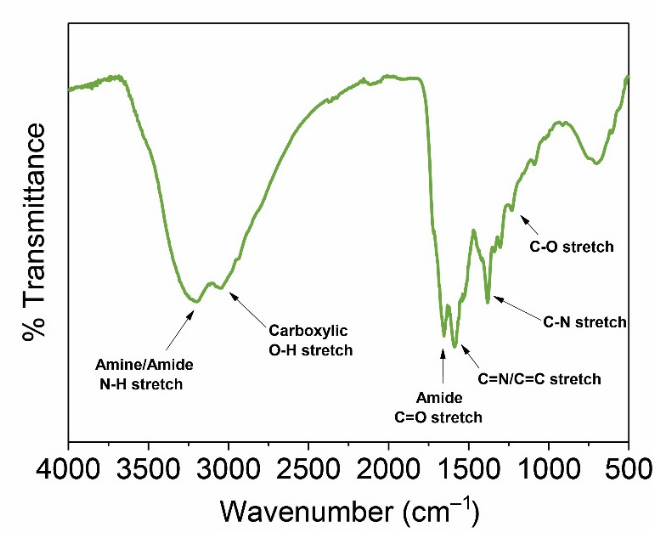


**Figure S2:** FTIR spectrum of FG-CDs showing the presence of amide and carboxyl stretches as well as N-H and O-H functional groups.

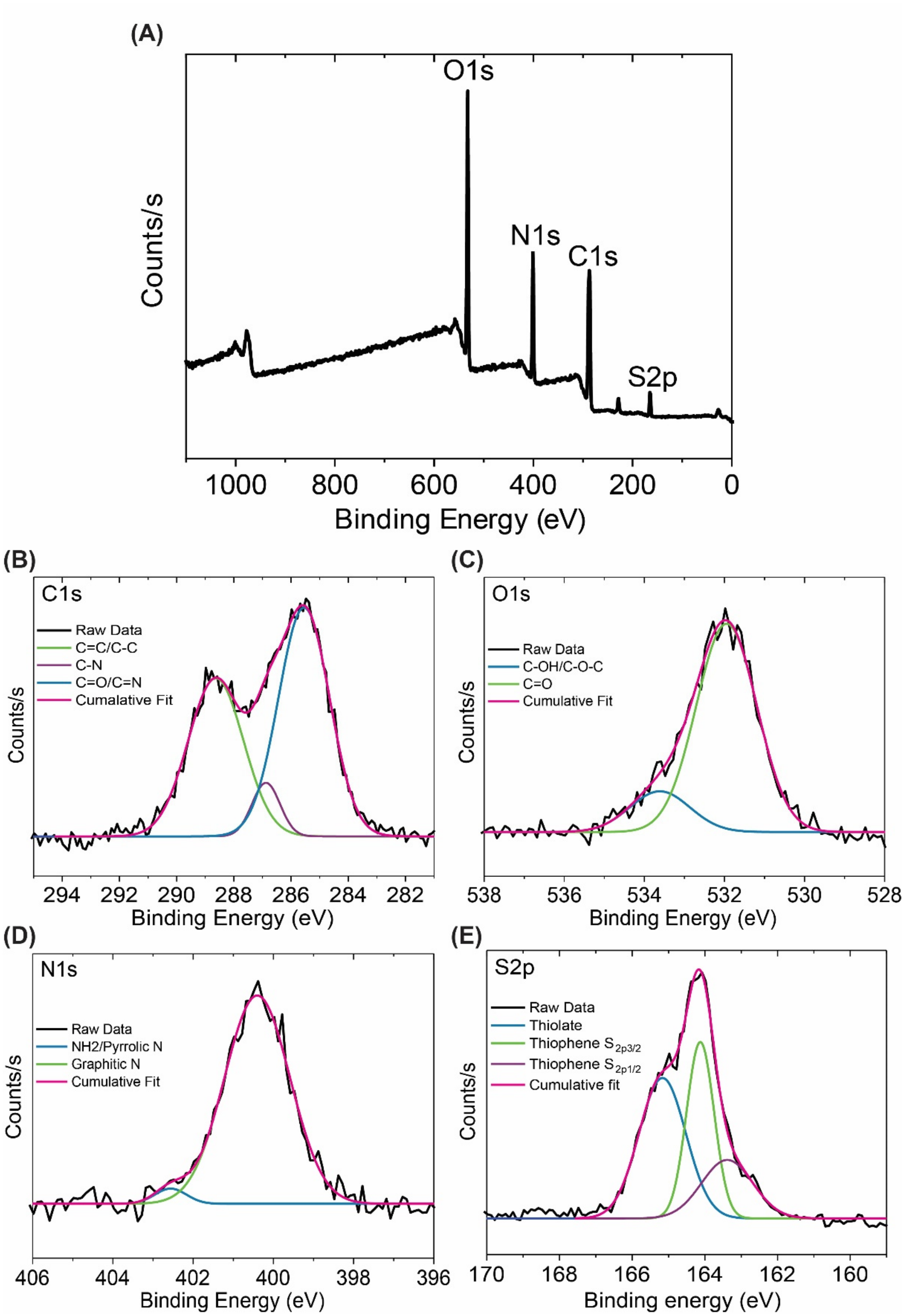


**Figure S3: (A)** XPS survey spectrum of FG-CDs showing binding energies of C1s, N1s, O1s and S2p. Spectra of deconvoluted binding energies reveal **(B)** a maximum for C1s at 286.08 eV, **(C)** a maximum at 400.08 eV for N1s **(D)** a maximum at 532.08 eV for O1s and **(E)** for S2p a maximum at 165.08 eV.

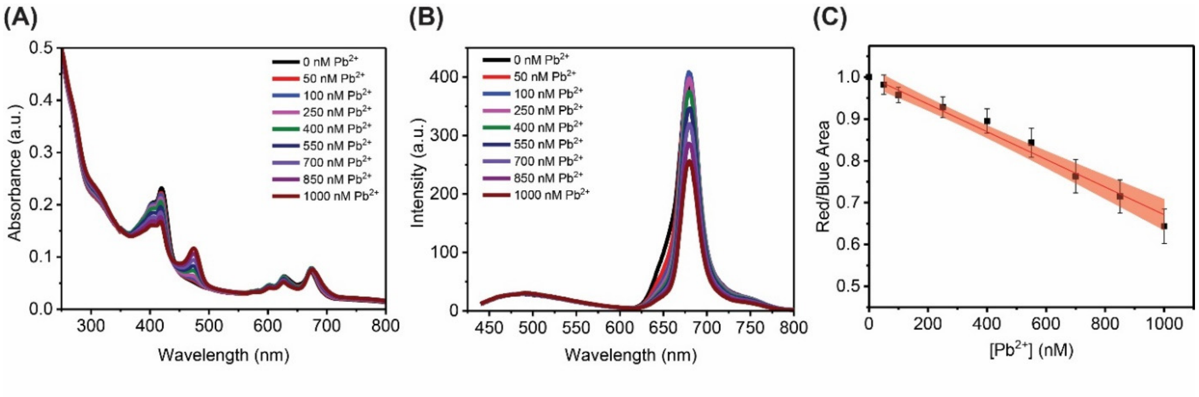


**Figure S4**: **(A)** Absorbance spectra for FG-CDs showing a new peak at ~475 nm in the presence of $Pb^{2+}$. **(B)** Fluorescence spectra for FG-CDs in the presence of $Pb^{2+}$. **(C)** The linear plot of the decreasing overall R/B area ratio showing a ~40% in red fluorescence with an $r^2$=0.98.

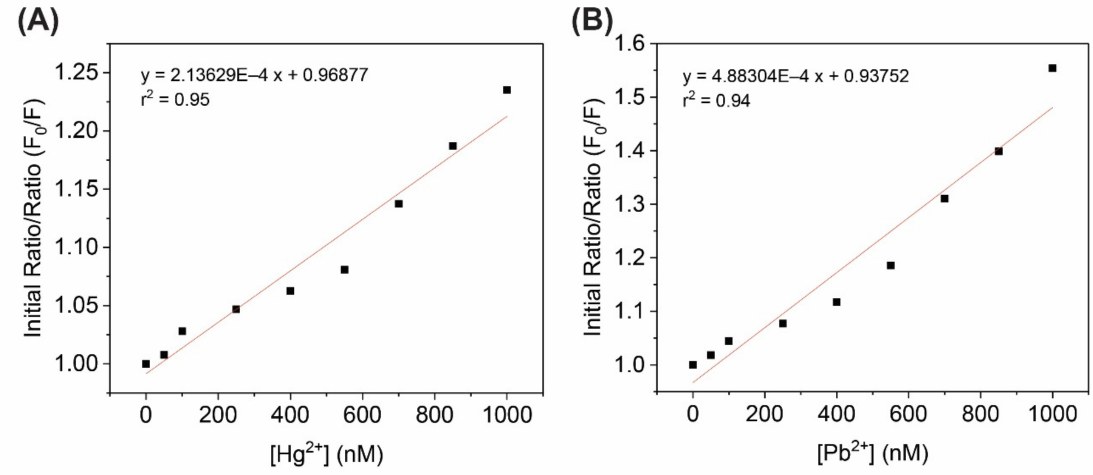


**Figure S5: (A)** Stern-Volmer plot displaying the linear equation for FG-CDs and $Hg^{2+}$ with $k_{sv}$ = 2.14x10$^{-4}$ and $r^2$= 0.95 **(B)** Stern-Volmer plot displaying the linear equation for FG-CDs and $Pb^{2+}$ with $k_{sv}$ = 4.88x10$^{-4}$ and $r^2$ = 0.94.

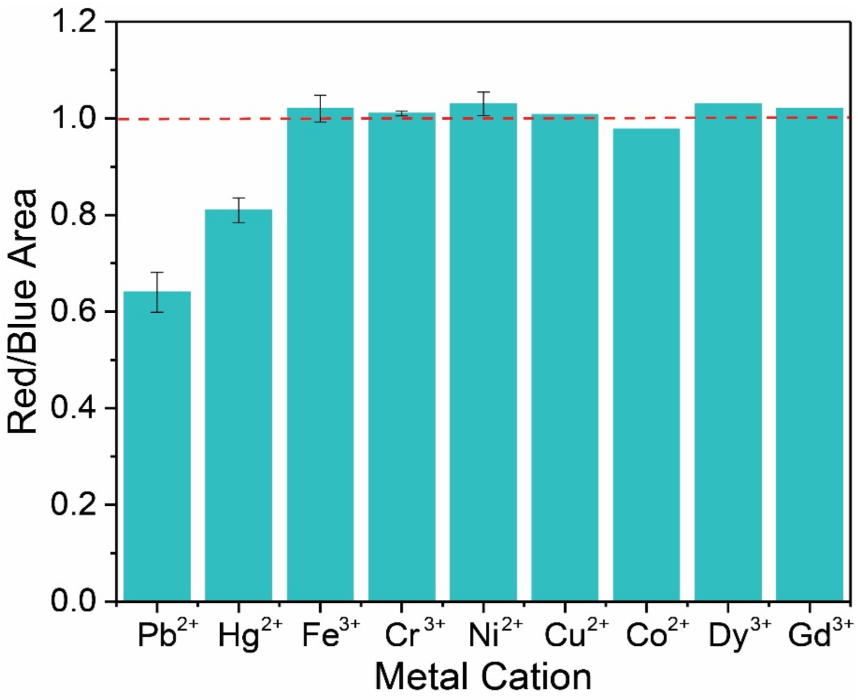


**Figure S6**: R/B area ratio of FG-CDs with 1000 nM of various metallic cations showing comparison of quenching effectiveness.
